# Detection of hereditary bisalbuminemia in bottlenose dolphins (*Tursiops truncatus,* Montagu 1821): comparison between capillary zone and agarose gel electrophoresis

**DOI:** 10.1186/s12917-016-0801-x

**Published:** 2016-08-20

**Authors:** Claudia Gili, Federico Bonsembiante, Renzo Bonanni, Alessia Giordano, Sabina Ledda, Giorgia Beffagna, Saverio Paltrinieri, Matteo Sommer, Maria Elena Gelain

**Affiliations:** 1 Costa Edutainment spa, Acquario di Genova, Area Porto Antico, Ponte Spinola, 16128 Genoa, Italy; 2 Department of Comparative Biomedicine and Food Science, University of Padua, AGRIPOLIS - Viale dell’Università 16, 35020 Agripolis, Legnaro, PD Italy; 3 Department of Veterinary Sciences and Public Health, University of Milan, Via Celoria 10, 20133 Milan, Italy

**Keywords:** *Tursiops truncatus*, Bisalbuminemia, Capillary zone electrophoresis, Agarose gel electrophoresis

## Abstract

**Background:**

Hereditary bisalbuminemia is a relatively rare anomaly characterized by the occurrence of two albumin fractions on serum protein separation by electrophoresis. In human medicine, it is usually revealed by chance, is not been clearly associated with a specific disease and the causative genetic alteration is a point mutation of human serum albumin gene inherited in an autosomal codominant pattern. This type of alteration is well recognizable by capillary zone electrophoresis (CZE), whilst agarose gel electrophoresis (AGE) not always produces a clear separation of albumin fractions. The aims of this study is to report the presence of this abnormality in two separate groups of related bottlenose dolphins and to compare the results obtained with capillary zone and agarose gel electrophoresis.

**Results:**

Serum samples from 40 bottlenose dolphins kept under human care were analyzed. In 9 samples a double albumin peak was evident in CZE electrophoresis while no double peak was noted in AGE profile. Since only an apparently wider albumin peaks were noted in some AGE electrophoretic profiles, the ratio between base and height (b/h) of the albumin peak was calculated and each point-value recorded in the whole set of data was used to calculate a receiver operating characteristic curve: when the b/h ratio of albumin peak was equal or higher than 0.25, the sensitivity and specificity of AGE to detect bisalbuminemic samples were 87 and 63 %, respectively. The bisalbuminemic dolphins belong to two distinct families: in the first family, all the siblings derived from the same normal sire were bisalbuminemic, whereas in the second family bisalbuminemia was present in a sire and in two out of three siblings.

**Conclusions:**

We report for the first time the presence of hereditary bisalbuminemia in two groups of related bottlenose dolphins identified by means of CZE and we confirm that AGE could fail in the identification of this alteration.

## Background

Bisalbuminemia, also called, alloalbuminemia, is a rare inherited or acquired serum protein abnormality characterized by the presence of two different peaks in the albumin fraction determined on serum protein electrophoresis. After electrophoretic screening of serum proteins, two distinct albumin bands or a single widened albumin band are evident [1]. In human medicine, the acquired or transient form is usually related to some drugs administration, such as high dose of β-lactamic antibiotics, as well as to different pathological conditions (e.g. multiple myeloma, pancreatic disease or neoplasia) [2]. On the contrary, hereditary bisalbuminemia is a relatively rare genetic anomaly, usually revealed by chance and is not been clearly associated with a specific disease. The causative genetic alteration is a point mutation of human serum albumin gene, inherited in an autosomal codominant pattern [3]. Albumin mutants (also called alloalbumins) are of interest because they are markers of migration and for population genetics, and because they can provide a model for the study of neutral molecular evolution [4].

The cumulative frequency of inherited bisalbuminemia is 1:1,000 to 1:10,000, with higher frequency found in isolated population or when a high resolution electrophoretic method was used [1, 5]. Since a single copy gene codominantly expressed synthesizes the protein, heterozygous subjects carrying point mutations usually show the presence of the normal and the variant proteins in a 1:1 ratio, with albumin variants exhibiting either an increased electrophoretic mobility (fast type variants) or decreased mobility (slow type variants), with total protein concentration remaining unchanged [6]. This type of alteration is well recognizable by capillary zone electrophoresis (CZE), whilst agarose gel electrophoresis (AGE) not always produces a clear separation of albumin fractions [7, 8] and it sometimes needs an increased migration time [6] and buffer pH [9]. In veterinary medicine, few reports describe the presence of bisalbuminemia in domestic or wild animals: this alteration is described in amphibians [10] and, recently, the presence of bisalbuminemia was reported in 4 healthy green iguanas [11]. As regards marine mammals, only two cases of bisalbuminemia were reported in apparently healthy bottlenose dolphins (*Tursiops truncatus*): the first case was a female, approximately of 10–12 years old, captured in 1977 in the Gulf of Mexico [12] and the second was a 10 years old male [13]. However, no data are reported about the presence of this electrophoretic pattern in related dolphins and, thus no information about the possible inheritance pattern of this condition is available.

Since bisalbuminemia was incidentally found during routine evaluation of health status in some dolphins kept under human care, we designed this study with the aims to report the presence this abnormality in two separate groups of related bottlenose dolphins of different origin and to compare the results obtained with CZE and AGE.

## Methods

Serum samples originated from 40 bottlenose dolphins with normal clinical history and physical examination; 21 males and 19 females (median age: 18 years, min-max: 1–51) maintained at Acquario di Genova (*n* = 14), Oltremare in Riccione (*n* = 11), Mediterraneo Park Malta (*n* = 8) and Zoomarine Italy (*n* = 7). Peripheral blood samples were obtained from individual animals during the veterinary procedures to evaluate health status of the animals where routine diagnostic tests, including hematology and serum biochemistry, also showed no abnormalities. The animals were housed and handled in agreement with the Italian and Maltese Zoo directive law (DL 73/2005 & S.L.439.08 respectively) and all the samples were obtained according to the D.M. 469/2001, which establishes the management objectives and prescriptions to maintain the species *Tursiops truncatus* under human care.

Blood was collected in plain tubes; serum was obtained by centrifugation of blood samples at 1500 g × 10 min. All serum samples were visually inspected and were not grossly hemolyzed or lipemic; thus, all samples were stored at -20C° until analysis.

All samples were analyzed by CZE and all but two were analyzed by AGE. Agarose gel electrophoresis was performed as already described [14] using an automated system and kits provided by the manufacturer of the instrument (Sebia Italia Srl, Bagno a Ripoli, Firenze, Italy). Briefly, a 0.8 % agarose gel was run in Trisbarbital buffer at pH 8.5 ± 0.3, with migration time of 7 min at 800 V. Gels were stained with amido Schwarz, destained, and dried for scanning by the appropriate gel scanner. Data were then transferred to the software program and visually inspected to correct the possible errors in fractions separation generated by the automated software (Phoresis, Sebia Italia Srl).

Capillary electrophoresis was performed with the MINICAP system 6 kit by SEBIA (Sebia Italia Srl), designed for the separation of serum protein in alkaline buffer (pH 9.9) into six major fractions. The MINICAP performs all analysis automatically to obtain a protein profile for qualitative and quantitative analysis. A sample dilution with buffer is prepared and injected by aspiration at the anodic end of the capillary. A high voltage protein separation is then performed and direct detection of the proteins is made at 200 nm at the cathode end of the capillary. The instrument records the absorbance corresponding to each electrophoretic fraction and send the data to the software (Phoresis, Sebia Italia S.r.l), which converts the absorbances in peaks. The capillaries are immediately washed with a wash solution and prepared for the next analysis with buffer. The electrophoretograms were interpreted visually to screen for any pattern abnormality.

For all samples, total protein (TP) concentration was also determined by the biuret method on an automated spectrophotometer (Cobas Mira, Roche Diagnostics, Basel, Switzerland), and absolute values for each electrophoretic fraction were calculated based on total protein and percentage of the fraction.

Electrophoretic profiles obtained with CZE and AGE were firstly visually analyzed to identify the presence of a double peak in the albumin region. Since only an apparently wider albumin peaks were noted in some AGE electrophoretic profiles, the ratio between base and height (b/h) of the albumin peak was calculated and each point-value recorded in the whole set of data was used to calculate a receiver operating characteristic (ROC) curve and to define the “ideal” cut-off value able to identify this abnormality in AGE electrophoresis. For this analysis, the true positive samples were considered those with a double peak in CZE.

The difference in total protein, albumin percentage and absolute values between bisalbuminemic and normal animal was evaluated by Mann–Whitney test while the differences between the albumin percentage obtained by CZE and AGE were analyzed with a Wilcoxon signed-rank test for paired data while. All statistical analysis was performed using standard statistical software (IBM® SPSS Statistics 22.0).

## Results

Total protein, as well as albumin percentage and absolute values obtained with both methods are reported in Table 1.

**Table 1:** Values of total protein and albumin in bisalbuminemic and normal bottlenose dolphins

		CZE tot *N* = 40		AGE tot *N* = 38	
	TP (g/L)	Albumin (%)	Albumin (g/L)	Albumin (%)	Albumin (g/L)
Bisalbuminemic (*N* = 9)	66.5 ± 7.1 (68.3; 50.8–74.0)	64.1 ± 3.4* (64.1; 58.6–68.1)	42.4 ± 3.70* (43.2; 33.7–46.1)	70.1 ± 3.6 (69.9; 64.6–76.0)	45.5 ± 3.6 (43.2; 38.6–49.4)
Normal	64.7 ± 5.8 (64.9; 48.0–72.7)	63.3 ± 3.3** (62.9; 57.7–72.0)	40.7 ± 3.4** (41.3; 30.9–45.9)	69.3 ± 3.4 (69.2; 61.0–77.3)	45.1 ± 4.0 (45.0; 36.9–54.1)
Tot	65.0 ± 6.0 (66.5; 48.0–74.0)	63.4 ± 3.3** (63.5; 57.7–72.0)	41.0 ± 3.5** (41.7; 30.0–45.9)	69.5 ± 3.4 (69.5; 61.0–77.3)	45.2 ± 3.9 (45.0; 36.9–54.1)

Values showed represent mean, standard deviation (median and min-max values) of total protein (TP), albumin (percentage and absolute values) obtained using capillary zone electrophoresis (CZE) and agarose gel electrophoresis (AGE)

*P < 0.05 vs albumin percentage and absolute value measured with AGE, **P < 0.001 vs albumin percentage and absolute value measured with AGE

**Fig. 1:**
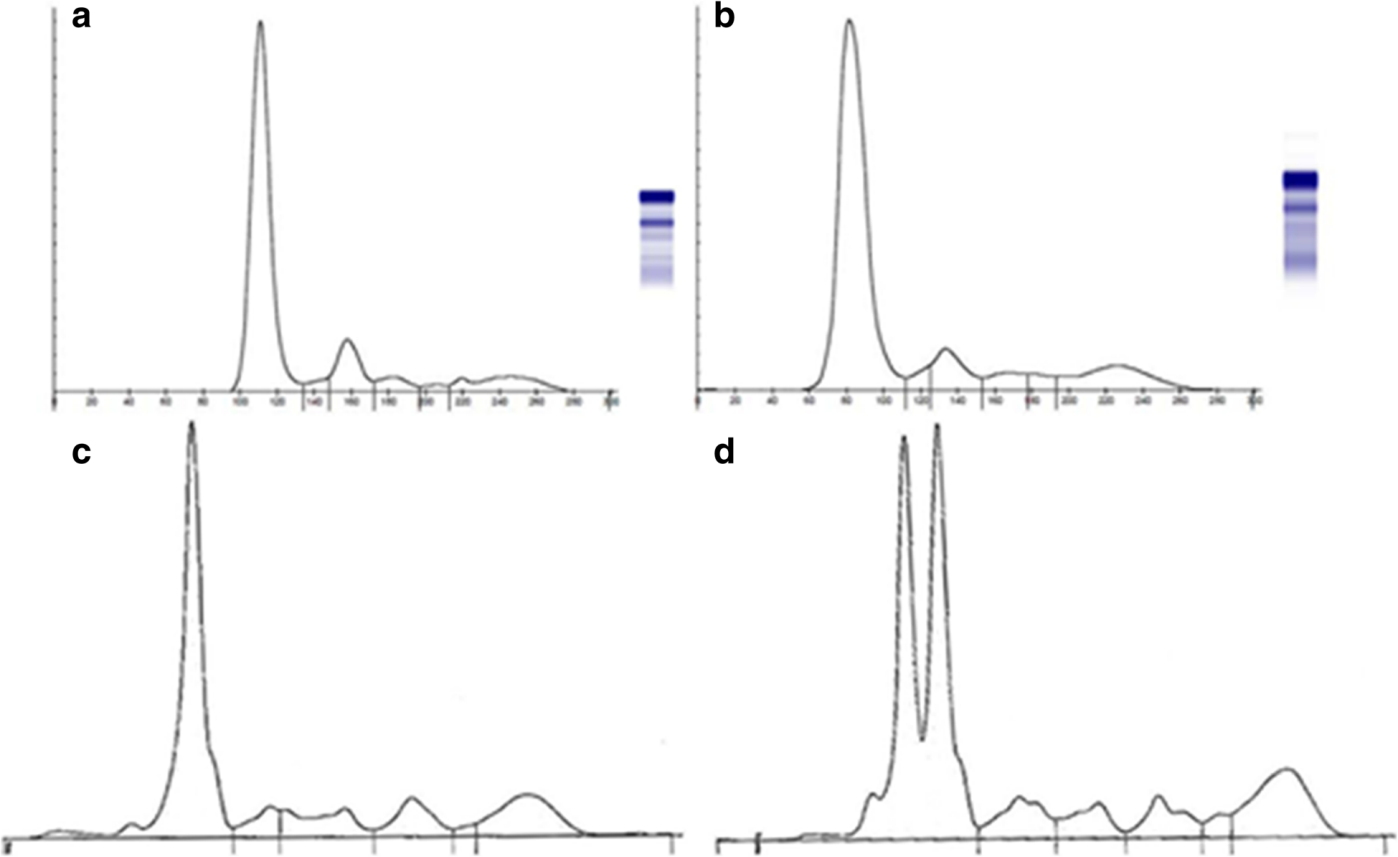
electropherogram of serum sample of unaffected and bisalbuminemic bottlenose dolphins. **a** Agarose gel electropherogram of serum sample of unaffected bottlenose dolphins, showing no abnormality, and **b** serum sample from bisalbumenic bottlenose dolphins, with a wider albumin band compare to normal one. **c** Capillary zone electropherogram of serum sample of unaffected bottlenose dolphins and **d** from bisalbumenic bottlenose dolphins, with a double albumin peak

At the visual examination, no double peak was noted in AGE profile while 9 out 40 samples (22 %) showed a double albumin peak in electrophoretic profiles obtained with CZE. However, all these samples except 2 had an albumin peak wider than that observed with AGE in dolphins classified as non bisalbuminemic by the CZE (Fig. 1); furthermore a wider albumin peak was also noted with AGE in one sample with normal CZE profile. The ROC curve analysis showed that when the b/h ratio of albumin peak was equal or higher than 0.25, the sensitivity and specificity of AGE to detect bisalbuminemic samples were 87 and 63 %, respectively with a AUC (area under the curve) of 0.773.

TP levels, albumin percentage and absolute values were not significantly different between normal and bisalbuminemic dolphins, but the albumin percentages obtained with CZE were significantly lower compared to AGE, either considering the whole set of data (*P* < 0.001) and only bisalbuminemic samples (*P* = 0.012) (Table 1).

**Fig. 2:**
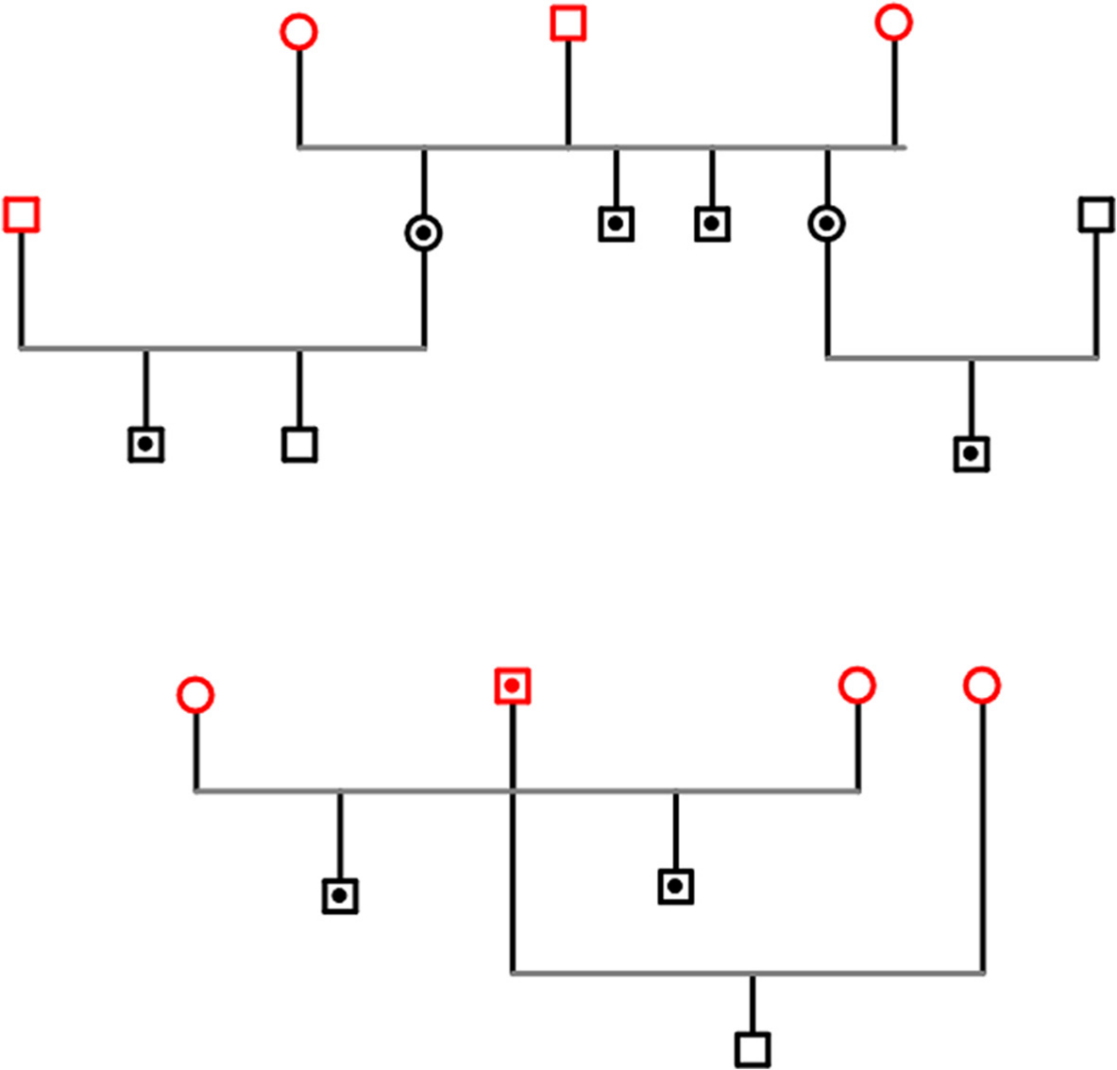
Pedigree of two groups of related bottlenose dolphins. Females are indicated by circles, males by squares. A black circle indicates affected dolphins, a white symbols illustrates unaffected dolphins. Red symbols indicate wild founders

The bisalbuminemic dolphins belong to two distinct families (Fig. 2), they were both male and female animals, and this phenotype was evident both in parents and offspring. In the first family, all the siblings derived from the same normal sire were bisalbuminemic, whereas in the second family bisalbuminemia was present in a sire and in two out of three siblings.

## Discussion

In the present work, we report for the first time the presence of hereditary bisalbuminemia in two groups of related bottlenose dolphins identified by means of capillary zone electrophoresis and we confirm that agarose gel electrophoresis could fail in the identification of this alteration, as already reported in human medicine [7, 8].

Serum protein electrophoresis is the most reliable method to determine the distribution of serum protein fractions and is considered, together with a basic hematological and biochemical profile, an essential step to evaluate the health status of animals, providing clinically useful information. The interpretation of kinetics of total proteins and albumin and globulin fractions is receiving increased attention also in marine mammals in which, as in terrestrial mammals, a typical pathologic pattern could be identified in several diseases, such as inflammatory diseases [15]. Nowadays, in many veterinary laboratories, CZE has replaced classical agarose gel electrophoresis, due to its higher resolution. The difference in resolution is mainly due to the different analytic method: while, in AGE, proteins migrate toward anode in a solid phase and in an alkaline buffer with low voltage, in CZE proteins rapidly move in a liquid phase toward the cathode thanks to the high voltage applied. This allows a better separation of proteins with similar physicochemical characteristics, thus generating multiple sub-peaks or narrower peaks [16]. When CZE was introduced routinely in human medicine laboratories, an increased number of bisalbuminemia cases was detected [7], based on the improved separation of the albumin, α1-globulin, and α2-globulin fractions. In our work, this technique clearly identified a double albumin peak at the visual analysis of the electrophoretic profile in 9 samples whereas with AGE only 8 profiles revealed a wider peak compared to the normal ones, but never an albumin double peak was detected. Furthermore, with AGE, an albumin peak apparently wider than normal was noted also in one sample with normal CZE profile, demonstrating as the visual interpretation of AGE profiles could lead to both false negative and false positive detection of bisalbuminemia. However, the visual identification of a “wider” peak could be considered a subjective method. Thus, we calculate the ratio between the length of the base and the height of the albumin peak with the aims to define a cut-off and to established a more accurate and objective method to identify bisalbumenimia in AGE electrophoretic profile. However, also with this approach the diagnostic accuracy was fair with a low specificity.

As expected, no significant differences in TP concentration, albumin percentage and absolute values between affected and normal dolphins was noted, but CZE albumin were significantly lower compared to AGE. In literature, an opposite situation is reported with higher albumin values obtained with CZE in dogs and cats [14]. Nevertheless, it’s possible that these data, despite the significant differences, are not clinically relevant. In literature, reference ranges for TP and serum protein fractions are available for free ranging bottlenose dolphins [17, 18]: compared to our results obtained with both methods, in free-ranging dolphins TP seemed higher and albumin absolute values lower, suggesting a higher concentration of globulins in these animals, thus a tendency to an inflammatory status, as already suggested [15]. All these data highlight the need to define appropriate reference ranges for different electrophoretic methods for bottlenose dolphins under human care.

The application of higher resolution techniques, such as CZE, can result in an increased number of “abnormal” profiles and thus a deeper knowledge of the clinical importance of these new profiles is required. In previous works on CZE validation in companion animals, an unusual albumin peak was observed in sera from clinically healthy cats never been reported previously, likely because of the low resolution of traditional electrophoretic techniques [14]. Thus, the correct interpretation of these kinds of data is essential to differentiate normal to pathological conditions. In human medicine, the diagnostic implications of the presence of bisalbuminemia in clinical entities are uncertain: it could be a sign of acquired disorders and it is correlated with several pathological conditions, like pancreatic and hepatic diseases (pancreatitis, pancreatic pseudocysts, hepatic chirrosis), lymphoproliferative diseases (monoclonal gammopathy, multiple myeloma) [2]. In our case, all the sampled dolphins were clinically healthy, without any other alteration in hematological or biochemical parameters and bisalbuminemia was detected accidentally, during the routine evaluation of the health status. Furthermore, no drugs were administered to the affected animals, except for an integration of folic acid in two of them, thus we could exclude the possibility that the abnormal electrophoretic pattern identified is due to a pathological condition or drugs administration.

The inherited form of human bisalbuminemia is usually discovered by chance and apparently does not seem associated with pathological conditions. Genetically, bisalbuminemia is due to a mutation in the albumin gene transmitted as an autosomal codominant trait and it has been reported in various human populations around the world, with significant differences in frequency in terms of race and location, with higher incidence in small, isolated population groups [3]. All the dolphins sampled in our study were living under human care and, from a reproductive point of view, they include wild founders and their progeny of first and second generation maintained in four groups separated in different facilities; for this reason the high incidence of this inherited disorder is somehow concentrated and not surprising.

Since the protein synthesis is governed by a single copy gene codominantly expressed, heterozygous subjects carrying point mutations usually show the presence of the normal and the variant proteins. Based on the pedigree of affected dolphins, we could only suppose the same inheritance pattern in bottlenose dolphins, but the molecular analysis of the albumin gene in affected dolphins and their related normal animals should be carried out to investigate the genetic defect underlying and the inheritance mode of transmission.

Over the last three decades, more than 60 different albumin variants have been characterized in people, being the vast majority reflecting single-base changes in the structural gene mainly with mutations in hypermutable CpG dinucleotides [1]. Rarely, the presence of bisalbuminemia may have a clinical impact due to the effect of mutation on ligand-binding: three mutations (p.Leu90Pro; p.Arg242His; and p.Arg242Pro) form strong binding sites for triiodothyronine (T3) or thyroxine (T4), causing the familiar dysalbuminemic hypertriiodothyroninemia, and the familiar dysalbuminemic hyperthyroxinemia syndromes [19, 20]. Other mutations seem to increase the binding capacity of long-chain fatty acids, but without clinical consequences [21]. Apparently, no signs of altered hormone or lipid binding capacity were evident in sampled dolphins based on the absence of laboratory abnormalities, but molecular information on genetic variants and mutations are needed to obtained valuable data about albumins binding properties.

## Conclusion

In conclusion, in this paper we reported the first description of inherited bisalbuminemia in 2 distinct families of bottlenose dolphins identified by CZE. Further studies will be needed in order to identify the causative genetic defects on albumin gene and a possible genotype-phenotype correlation, in particular regarding consequences on albumin affinity for endogenous or exogenous ligands.

## Abbrevations

AGE: Agarose gel electrophoresis
CZE: Capillary zone electrophoresis
ROC: Receiver operating characteristic
TP: Total protein
